# Treatment of Open Bite Based on Skeletal Anchorage Using Extrusion Lever Arms and Class III Elastics

**DOI:** 10.1155/2024/7768109

**Published:** 2024-04-08

**Authors:** Miodrag Popov, Nemanja Marinkovic, Ivan Arsic, Predrag Janosevic, Nenad Nedeljkovic

**Affiliations:** ^1^Clinic for Orthodontics, School of Dental Medicine, University of Belgrade, Belgrade, Serbia; ^2^Clinic for Dental Medicine, Dental Department, Medical Faculty, University of Nis, Nis, Serbia

## Abstract

A 26-year-old woman had a masticatory dysfunction, straight profile, retrognathic maxilla and mandible, and Angle's class I with a tendency to class III malocclusion on both sides, with bilateral posterior crossbites and a 4 mm anterior open bite. Orthognathic surgery and orthodontic camouflage with and without tooth extraction were considered as treatment options. The patient's preferred method of treatment was orthodontic camouflage without extraction. The transpalatal arch had been placed for the bilateral molars' derotation. After 3 months, the upper segmented fixed appliance was implanted to address the posterior crossbites in the premolar's region. One mini-implant was inserted into the anterior palatum after the transpalatal arch was removed, and a supporting device was attached to the first permanent molars to give indirect skeletal stability. Orthodontic treatment's active phase lasted 23 months, and all treatment objectives were achieved during that time: the desired facial profile, adequate occlusion, appropriate overbite, and overjet.

## 1. Introduction

Open bite is defined as the absence of occlusal contact in the vertical direction between the teeth of the opposing dental arches at the maximum intercuspation [1, 2]. The lack of occlusal contact can be located in the anterior or posterior region of dental arches, caused by numerous external, environmental, anatomic, and genetic factors [3].

The treatment objectives of open bite malocclusion are to improve facial esthetics and harmony and to establish normal performance of all oral functions [4]. For adults, orthodontic compensation and orthognathic surgery can be considered for skeletal open bite treatment; however, no preferred treatment method with a high success rate has been determined yet [5]. The use of mini-implants has enabled a wider range of sagittal and vertical skeletal discrepancies that could be corrected without orthognathic surgery [6].

The anterior open bite represents one of the greatest treatment challenges in orthodontics due to high long-term relapse rate [7]. The prevalence of anterior open bite varies up to 11% in mixed and up to 8.7% in permanent dentition, depending on the nation in which the research was conducted [8]. Hyperdivergent skeletal pattern, lip incompetence, maxillary constriction with posterior crossbite, mandibular crowding, and class II malocclusion are usual characteristics of skeletal anterior open bite [9]. A combination of orthodontic treatment and orthognathic surgery has been the most frequently recommended therapy for patients with skeletal anterior open bite [10]. Recently, open bite treatment option in adult patients could be a molar intrusion, which is accomplished through the use of fixed orthodontic appliances, mini-implants, clear aligners, rapid molar intruders, and corticotomy-assisted intervention [11]. However, the intrusion of posterior teeth causes counterclockwise rotation of the mandible and advancement of the chin which can improve a class II patient's profile and deteriorate a class III patient's profile from straight to an esthetically less satisfying concave profile [12].

The article describes how fixed appliances and one mini-implant were used to treat the severe anterior open bite without tooth extraction and orthognathic surgery in the adult patient with a bilateral posterior crossbites and a tendency to class III malocclusion.

## 2. Case Report

### 2.1. Diagnosis and Etiology

Masticatory dysfunction was the main complaint of a 26-year-old female who visited the clinic for orthodontics for consultation. After the patient completed the medical questionnaire, it was confirmed the absence of systematic disorders, significant head trauma, previous orthodontic treatment, and similar orthodontic anomaly in close relatives.

**Figure 1:**
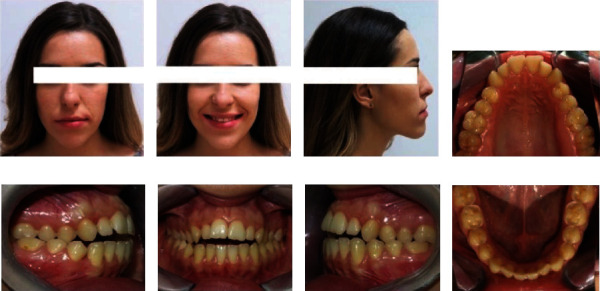
(a–c) Extraoral and (d–h) intraoral photography before orthodontic treatment.

According to the extraoral inspection (Figure 1), she had an almost symmetrical face, increased lower face height, a straight profile with a slightly prominent chin, and acceptable contact between the lips in rest position. Only the incisal third of the crowns of the upper anterior teeth was visible when the patient smiled.

**Figure 2:**
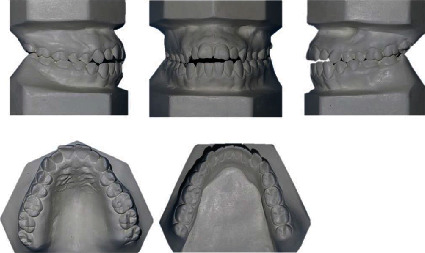
Dental casts before orthodontic treatment.

The intraoral examination showed Angle's class I with a tendency to class III malocclusion on both sides, with bilateral posterior crossbites and a 4 mm anterior open bite. The facial midline corresponded with the maxillary and mandibular midlines. There were no clinical manifestations or noticeable symptoms of temporomandibular joint disorder. At rest, the patient's tongue was in an anterior position, and she swallowed with a tongue-trust pattern. The analysis of dental casts reported that both the maxillary and mandibular arch had a 5 mm and 3 mm space discrepancy, respectively (Figure 2).

**Figure 3:**
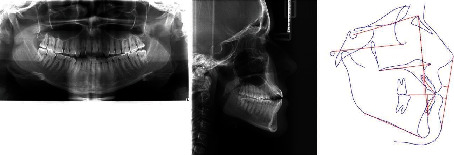
Initial panoramic and lateral cephalometric radiography.

The panoramic radiograph revealed that all third molars were fully developed, that cavity fillings were present on some teeth in both jaws, and no radiologically evident signs of pathological processes in the bone or periapical tissue (Figure 3). The lateral cephalometric analysis showed maxillary retrognathism followed by mandibular retrognathism, tendency to skeletal class III relationship (ANB = 1°) associated with excessive vertical, and hyperdivergent growth pattern (FMA = 29°). Both the maxillary and mandibular incisors were proclined (Table 1).

### 2.2. Treatment Objectives

Treatment objectives were to achieve acceptable vertical and horizontal overlap of anterior teeth, molar and canine relationships in class I, and an adequate transversal relationship of posterior teeth, to improve smile esthetic with increasing incisor exposure and not aggravate the existing facial profile.

### 2.3. Treatment Alternatives

Treatment options, both orthognathic surgery and orthodontic camouflage, were considered. The first treatment option consists of two phases. Presurgical orthodontic treatment to align and level the teeth in both dental arches would be the first phase. The second phase consists of a comprehensive surgical intervention to correct skeletal sagittal and vertical discrepancies between the maxilla and mandibulae. In order to address the vertical and sagittal irregularities, orthodontic camouflage would entail extracting the lower first premolars, applying fixed orthodontic appliances, and using class III and vertical intermaxillary elastics. The third nonextraction treatment option includes correcting the position of rotated maxillary molar, segmental fixed appliance in the maxilla, a mini-implant on the palate providing skeletal absolute anchorage, and later, fixed appliances, extrusion of upper anterior teeth, and the use of intermaxillary class III elastics. The proposed option would be an alternative solution that may not give the ideal results that orthognathic surgery could achieve; however, the patient's masticatory function would be improved. After careful evaluation of all the benefits and drawbacks of all treatment options, the patient chose a less invasive third alternative option, instead of surgery and extraction.

### 2.4. Treatment Progress

**Figure 4:**
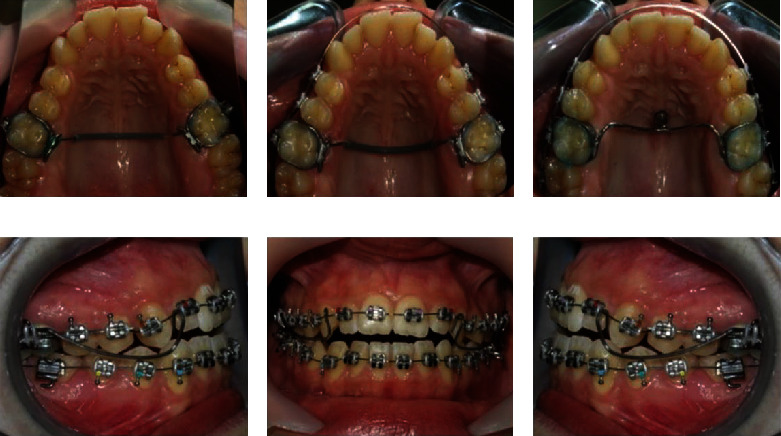
(a) Placement of the transpalatal arch. (b) Added upper segmented fixed appliance. (c) Supporting mechanism connected with palatal mini-implant that enabled indirect skeletal anchorage. (d) Right profile. (e) En face and (f) left profile intraoral photography of upper and lower fixed appliance with beta-titanium cantilever.

In the first phase of treatment, the bands were cemented on the first permanent molars, and the transpalatal arch was placed, which was activated for bilateral molar derotation (Figure 4(a)). Class III molar relationships with corrected buccolingual relations were additionally emphasized after molar derotation. After 3 months, the upper segmented fixed appliance was inserted, which included the upper premolars in addition to the first permanent molars (Figure 4(b)). The segmental fixed appliance was installed to treat the posterior crossbites in premolar region. The leveling was started with rounded initial archwire 0.016-inch nickel-titanium (Ni-Ti), and it has been used in 0.022 slot brackets (Roth prescription). The leveling procedure was carried out on a monthly basis up to a stainless steel wire (SS) 0.019 × 0.025 inch (the duration of this phase was 5 months).

The transpalatal arch was removed in the next stage, and one mini-implant was placed in anterior palatum with a supporting device attached to the first permanent molars to provide indirect skeletal anchorage (Figure 4(c)). The upper and lower fixed appliance was inserted in the same time with rounded initial archwire 0.014-inch Ni-Ti. Class III elastics (2 oz; size 5/16 inches) were immediately incorporated to achieve uprighting and distalization of the lower posterior teeth and extrusion and retroinclination of the lower incisors (Figures 4(d)–4(f)). For extrusion of the upper front teeth and correcting the mesially inclined canine, we applied on mesial and distal side the extrusion lever arms composed of beta-titanium (TMA) wire, 0.017 × 0.025 inch (force dosed 30 gr per side) (Figures 2(d)–2(f)).

The next archwire that we used was 0.016-inch Ni-Ti, and the patient continued to use intermaxillary class III elastics (3 oz; size 1/4) that was extended from the lower canine to the upper first molar. The archwire 0.016 × 0.022-inch Ni-Ti with intermaxillary elastics class III (4 oz; size 3/16) was the following step in the leveling procedure. The patient was instructed to swap out old elastics for new ones each day. The orthodontic treatment was completed on a SS wire 0.019 × 0.025 inch in both dental arches.

### 2.5. Treatment Results

**Figure 5:**
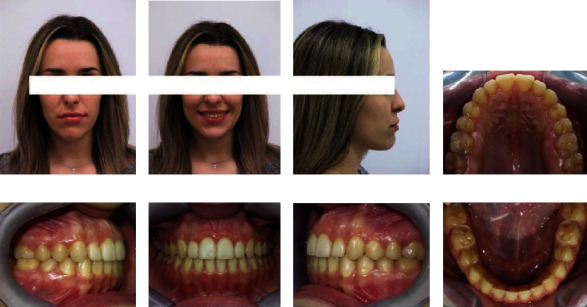
(a–c) Extraoral and (d–h) intraoral photography after orthodontic treatment.

When all treatment goals were met, the active phase of orthodontic therapy lasted 23 months. The upper first permanent molars were derotated in the first three months of orthodontic treatment. In addition, the bilateral crossbites in the lateral tooth region were corrected. The buccal corridors, which were visible when the patient smiles, were significantly reduced after the correction of transverse occlusal relations, which improved the esthetics of the patient's smile (Figure 5).

If the position of the cephalometric point pogonion was observed before and after orthodontic treatment, it can be noticed that the pogonion was moved slightly backwards as a result of the action of class III elastics and the change of angle between the occlusal plane and the anterior cranial plane (NS). At the end of the orthodontic treatment, we could see that the profile was slightly flattened and improved. Extrusion of the upper and lower permanent incisors occurred which allowed the bite to close. On the other hand, extrusion of the incisors provided their greater visibility when smiling. Further, by extruding the incisors, the smile line has improved and now follows the contour of the lips.

**Figure 6:**
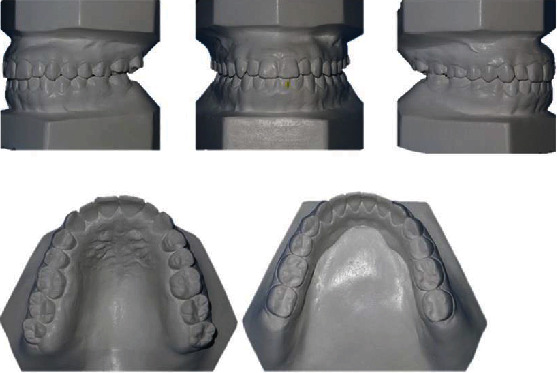
Dental casts after finishing the orthodontic treatment.

**Figure 7:**
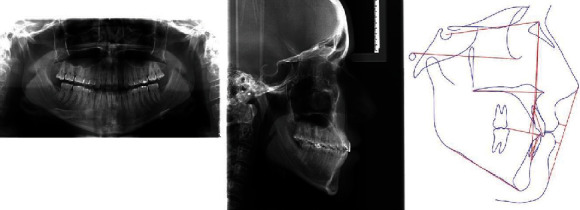
Panoramic and lateral cephalometric radiography after the orthodontic treatment.

Figures 6 and 7 show dental casts, panoramic, and lateral cephalometric radiography obtained after orthodontic treatment.

## 3. Discussion

In adult patients, general treatment to achieve bite closure entails a combination of fixed orthodontic appliances and orthognathic surgery [13]. Skeletal anchorage devices have been developed and proposed as an alternative option to orthognathic surgery for treating a skeletal open bite [13].

Since our patient had mild to moderate crowding in both the upper and lower dentition, we decided to proceed with orthodontic treatment without extracting any teeth. In camouflage treatment of patients with class III malocclusion and anterior open bite, tooth extractions are often needed and most commonly occur in the lower dental arch [14]. Although extraction would enhance the vertical component of the patient's occlusion, due to the anatomical and morphological characteristics of the edentulous alveolar ridge, closing the remaining extraction space could be difficult, time-consuming, and possible inefficient.

One of the treatment strategies for closing the bite is molar intrusion with mini-implants. However, molar intrusion is often accompanied by a counterclockwise rotation of the mandibula, which might help patients with class II malocclusion to improve their convex facial profile [15]. In patients with class III malocclusion, this form of movement would make the patient's facial profile more concave, emphasizing the class III malocclusion [15]. On the other hand, clockwise rotation of mandibula would further aggravate the anterior open bite. In addition, incisor extrusion is a therapeutic alternative for anterior open bite. In several published case reports, the authors used two or four buccally placed mini-implants to achieve molar intrusion and bite closure [12, 16]. Our treatment plan involved for the placement of just one mini-implant in the palate's front region, which decreased treatment costs, improved circumstances for maintaining oral hygiene, and reduced the possibility of implant loss compared to buccal placement.

**Figure 8:**
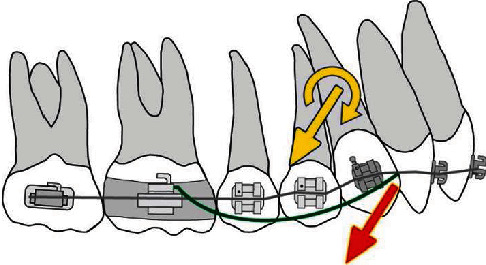
The effect of TMA cantilever.

The use of a beta-titanium extrusion lever arms enabled the application of the force at the level of the center of resistance of the upper anterior arch segment, which led to the bodily extrusion of the frontal teeth while correcting the canine mesial inclination (Figure 8).

The placement of vertical intermaxillary elastics in the anterior region would result in an extrusion with uncontrolled tipping of the anterior teeth during the initial stages of treatment (when thin nitinol archwire is placed), as the point of force application is located in front of the center of resistance (Figure 8).

**Figure 9:**
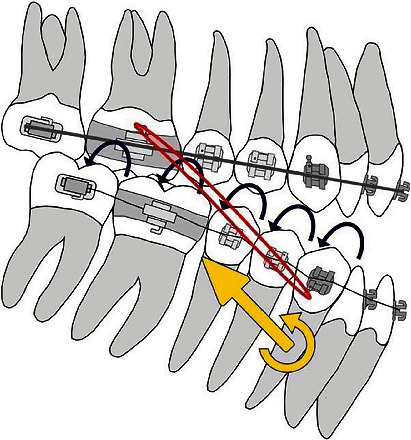
Biomechanics scheme of class III elastics with an indirectly connected first molar with a mini-implant.

The lower posterior teeth had mesial inclination, whereas crowding and proclination had been diagnosed in the region of anterior teeth. We used class III intermaxillary elastics extended from the lower canine to the upper first molar on thin rounded nickel-titanium archwire (0.014-inch Ni-Ti). A transpalatal arch laying on the one palatal mini-implant was stabilized the position of the first permanent molars in the maxilla. In addition to providing a strong anchorage for class III intermaxillary elastics and their impact on the lower dental arch, the use of one mini-implant in combination with a transpalatal arch allowed us to achieve the desired movement of the upper front teeth. Class III elastics applied in this situation will cause the distal tip back moment and the uprighting of the posterior teeth, which will provide space for resolving the crowding in the region of the lower anterior teeth (Figure 9). Likewise, the applied force caused the lower incisors to extrude, which contributed to the treatment of the anterior open bite.

**Figure 10:**
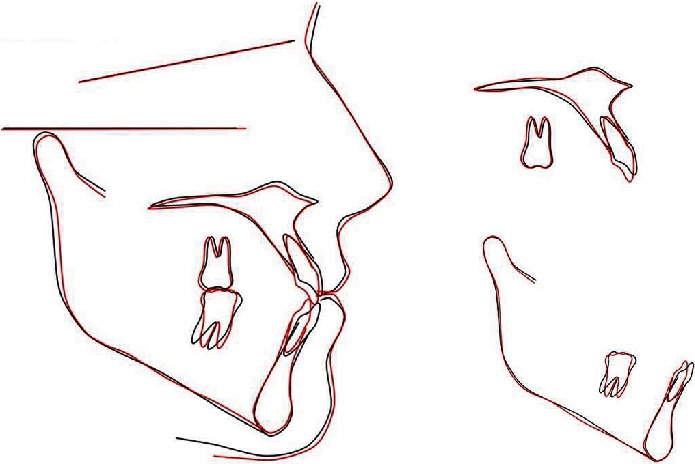
Superimpositions of pretreatment (black line) and posttreatment (red line) cephalometric tracings.

Extrusion of incisors was one of the goals of orthodontic treatment with fixed appliances, which resulted in increased anterior overbite and visibility of frontal teeth when smiling. Once the leveling phase is completed and a rectangular stainless steel arch-wire was applied, the lower dentition acts as a single body that moves counterclockwise around the center of resistance which is located in the region of the lower second premolar. This permitted the lower occlusal plane to be straightened and turned counterclockwise, bringing it into alignment with the upper occlusal plane. Described alignment of the upper and lower occlusal plane aided in closing the anterior open bite (Figure 10).

It has been demonstrated that when molar intrusion with mini-implants or orthognathic surgery was utilized to close the anterior open bite, in 10 to 30% cases, the relapse was occurred [17]. The main reason for relapse was altering the vertical position of the upper or lower molars [17]. This indicates that, whenever possible, mini-implants should be preferred over surgical procedures in the treatment of the anterior open bite, as they have a similar recurrence rate as surgical procedures [18, 19]. Likewise, mini-implants are much more comfortable for patients and less expensive and have a shorter postoperative recovery period than surgical treatment [19].

**Figure 11:**
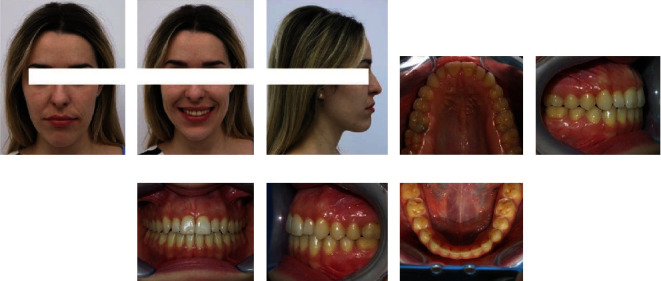
(a–c) Extraoral and (d–h) intraoral photography after 3 years of retention.

After completing the treatment, we provided the patient a removable Essix retainer that she wore according to a certain protocol [20]. Extraoral and intraoral photography after 3 years of retention are presented in Figure 11. The recent findings showed that extraction led to a higher level of stability of the outcomes, whereas nonextraction cases showed a lower level of stability [21]. However, we were able to keep the final result stabile. At the 3-year follow up, the occlusion on first molars and canine was in class I relationship with optimal overbite and overjet. The fact that the bite was not closed by rotating the mandible counterclockwise, but incisor extrusion and the parallelism of the upper and lower occlusal planes were achieved, which offered additional stability to solid tooth contacts in central occlusion, could be one reason for the results' stability. This might confirm the statement that the formulation of static and dynamic criteria is critical to the effectiveness of orthodontic treatment that could reduce orthodontic relapse and prevents developing abnormal occlusal relations [22].

## 4. Conclusions

This report documented the treatment of an anterior open bite with class III malocclusion and bilateral posterior crossbite without surgery procedures.

The use of palatal mini-implant, transpalatal arch, and cantilever TMA wire enabled straightening and extrusion of the incisors and simultaneous parallelism of the upper and lower occlusal planes, which led to the successful closure of the bite and improving the esthetics of the patient's face.

## Figures and Tables

**Table 1:** Cephalometric measurements before and after the treatment.

Parameter	Before treatment	After treatment
SNA (°)	78.8	79.5
SNB (°)	77.7	78.1
ANB (°)	+1.1	+1.4
AO-BO (mm)	−3.5	−5.7
Cant of occlusal plane (°)	5.4	8.5
Maxillary mandibular plane angle (°)	30.6	30.2
Bjork sum (°)	399.9	399.5
FMA (°)	29.2	28.5
FMIA (°)	56.5	67.2
IMPA (°)	94.3	84.3
U1 to maxillary plane angle (°)	119.8	110.5
Li-E (mm)	−4.1	−3.8
Ls-E (mm)	−6.4	−5.7
